# New Work on Southern Diseases

**Published:** 1843-06

**Authors:** 


### NEW WORK ON SOUTHERN DISEASES.

It gives us pleasure to learn that our correspondent, Dr. Dowler, of New Orleans, has quite ready for the press, a work on the diseases of the South, referring chiefly to fevers, bowel complaints, &c. From what we know of Dr. Dowler, we anticipate a production of much value, and shall look for its appearance with a good deal of interest. C.

